# High abundance of sugar metabolisers in saliva of children with caries

**DOI:** 10.1038/s41598-021-83846-1

**Published:** 2021-02-24

**Authors:** Muhammed Manzoor, Sohvi Lommi, Jussi Furuholm, Catharina Sarkkola, Elina Engberg, Sajan Raju, Heli Viljakainen

**Affiliations:** 1 Folkhälsan Research Center, Helsinki, Finland; 2 Department of Public Health, Faculty of Medicine, University of Helsinki, Helsinki, Finland; 3 Department of Oral and Maxillofacial Diseases, Faculty of Medicine, University of Helsinki, Helsinki, Finland; 4 Department of Psychology and Logopedics, Faculty of Medicine, University of Helsinki, Helsinki, Finland; 5 Faculty of Medicine, University of Helsinki, Helsinki, Finland

**Keywords:** Microbial communities, Pathogens, Sequencing, Diseases, Medical research, Risk factors

## Abstract

Dental caries is a biofilm-mediated, dynamic disease with early onset. A balanced salivary microbiota is a foundation of oral health, while dysbiosis causes tooth decay. We compared the saliva microbiota profiles in children with and without caries. The study consisted of 617 children aged 9–12 years from the Finnish Health in Teens (Fin-HIT) study with available register data on oral health. Caries status was summarised based on Decayed, Missing, and Filled Teeth (DMFT) index in permanent dentition. The children were then classified into the following two groups: DMFT value ≥ 1 was considered as cavitated caries lesions (hereafter called ‘caries’) (n = 208) and DMFT = 0 as ‘cavity free’ (n = 409). Bacterial 16S rRNA gene (V3–V4 regions) was amplified using PCR and sequenced by Illumina HiSeq. The mean age (SD) of the children was 11.7 (0.4) years and 56% were girls. The children had relatively good dental health with mean DMFT of 0.86 (1.97). Since sex was the key determinant of microbiota composition (*p* = 0.014), we focused on sex-stratified analysis. Alpha diversity indexes did not differ between caries and cavity free groups in either sexes (Shannon: *p* = 0.40 and 0.58; Inverse Simpson: *p* = 0.51 and 0.60, in boys and girls, respectively); neither did the composition differ between the groups (*p* = 0.070 for boys and *p* = 0.230 for girls). At the genus level, *Paludibacter* and *Labrenzia* had higher abundances in the caries group compared to cavity free group in both sexes (*p* < 0.001). Taken together, there were minor differences in saliva microbiota between children with and without caries. Potential biomarkers of caries were the sugar metabolisers *Paludibacter* and *Labrenzia*. These bacteria presumably enhance salivary acidification, which contributes to progression of dental caries. The clinical relevance of our findings warrants further studies.

## Introduction

Human saliva consists of diverse and numerous microorganisms including bacteria, fungi, viruses, archaea, and protozoa, commonly called the saliva microbiota^1^. The saliva microbiota is an important part of the human microbiome. Dysbiosis in the saliva microbiota is associated with various human diseases, including obesity^2^, inflammatory bowel diseases^3^, celiac disease^4^, Sjögren's syndrome^5^, and Kostmann syndrome^6^.

The oral cavity is a major route into the human body, and, therefore, the saliva microbiota is vital in maintaining both systemic and oral health^7^. Recent evidence suggests that the normal saliva microbiota protects against colonization of pathogenic bacteria, plays a fundamental role in maintaining oral homeostasis, and prevents development of various oral diseases, including dental caries^8–10^. In general, the saliva microbiota is considered a good indicator of oral health^11^.

Dental caries is a biofilm-mediated, dynamic disease with early onset and a significant oral health problem in humans worldwide ^12,13^. The prevalence of caries varies significantly between various parts of the world. A high prevalence is reported in many developing countries in Asia and Africa and also in some Central and Eastern European countries^14^. The aetiology of caries is complex and multifactorial and includes lifestyle factors (such as dietary habits, especially frequent consumption of dietary sugars), oral hygiene, use of antibiotics, fluorides, susceptible tooth surface, and biofilms^12^. In addition, recent evidence suggests that caries is a polymicrobial disease with some non-infectious species. However, increases in cariogenic microbial species result in tooth demineralization^9^. Moreover, caries is enhanced due to an ecological imbalance in the commensal microbes of the oral cavity, including sugar-fermenting and acidogenic bacteria, especially *Streptococcus mutans*^15^.

Several studies have reported that individual bacteria in saliva are positively associated with dental caries, including *Streptococcus*^16^, *Lactobacilli*^17^, and *Bifidobacteria*^18^. Recently, the association of saliva microbiota with dental caries has been examined in adults and in the elderly in Japan, Sweden, Germany, and China^19–22^. However, these results were not consistent and implied that the association is complex and likely depends on the population. Some pilot studies have suggested that alterations in the saliva microbiota correlate with caries development in children^23,24^. However, neither age nor sex was considered in the analysis; we, and others, have demonstrated that the composition of saliva microbiota is sex-specific in children^2,25,26^.

To the best of our knowledge, there have been no large-scale studies that compared saliva microbiota profiles in children with and without caries. Therefore, the present study sought to investigate the composition, diversity, and abundance of saliva microbial communities in Finnish children with and without caries history in permanent dentition. The study will provide a basis for a better understanding of the microbial aetiology of caries in children and serve as the foundation for novel therapeutic strategies for caries prevention.

## Results

### Characteristics of the study population

The present study included 617 participants. Of these, 33.7% had a history of caries; 36.8% of girls and 29.8% of boys had experienced caries based on the register data. Participants with a history of cavitated caries lesions (hereafter called ‘caries’) and no history of cavitated caries (hereafter called ‘cavity free’) were compared with respect to sex, age, body mass index (BMI) z-score, native language, Decayed, Missing, and Filled Teeth (DMFT) index, and oral hygiene risk (Table 1). Among these, native language (Finnish, Swedish or other) differed between caries and cavity free groups (*p* = 0.017). The proportion of girls appeared higher in the caries group than in the cavity free group, however, the difference was not statistically significant (61.1% [127/208] vs 53.3% [218/409]; *p* = 0.072). The average (standard deviation, SD) age of participants was 11.7 (0.4) years and ranged from 10.6 to 14.1 years. Median age was similar in the caries and cavity free group [caries 11.7 (0.3) vs cavity free 11.8 (0.4); *p* = 0.051]. In the entire group, the mean DMFT score was 0.86 (1.97) with minimum 0 and maximum 24. The maximum Community Periodontal Index for Treatment Needs (CPITN) value varied from 0 to 2, and 34.8% of the children had healthy gingival tissue (hereafter called ‘no oral hygiene risk’) with a maximum CPITN value of 0.

**Table 1:** Comparisons performed between caries and cavity free groups for whole sample and separately for boys and girls.

	Whole sample (N = 617)			Boys (n = 272)			Girls (n = 345)		
	caries (n = 208)	cavity free (n = 409)	*p*-value	caries (n = 81)	cavity free (n = 191)	*p*-value	caries (n = 127)	cavity free (n = 218)	*p*-value
**Age in years, median (SD)**	11.7 (0.3)	11.8 (0.4)	0.051^£^	11.7 (0.4)	11.7 (0.4)	0.302^£^	11.7 (0.4)	11.7 (0.3)	0.079^£^
**BMI z-score, mean (SD)**^**a**^	0.1 (1.0)	0.1 (1.0)	0.730^£^	-0.1 (1.0)	0.1 (1.1)	0.311^£^	0.2 (0.9)	0.1 (0.9)	0.269^£^
**Native language, n (%)**									
Finnish	174 (83.7)	354 (86.6)	0.017^#^	71 (87.6)	163 (85.3)	0.328^#^	103 (81.1)	191 (87.6)	0.007^#^
Swedish	20 (9.6)	46 (11.2)		5 (6.2)	21 (11)		15 (11.8)	25 (11.5)	
Other	14 (6.7)	9 (2.2)		5 (6.2)	7 (3.67)		9 (7.1)	2 (0.9)	
**Gingival health, n (%)**									
No risk	67 (32.2)	148 (36.2)	0.372^#^	24 (29.6)	53 (27.7)	0.430^#^	43 (33.9)	95 (43.6)	0.048^#^
Elevated risk	141 (67.8)	261 (63.8)		57 (70.4)	138 (72.3)		84 (66.1)	123 (56.4)	
**Alpha diversity indexes, mean (SD)**									
Shannon	2.92 (0.29)	2.92 (0.28)	0.613^£^	2.97 (0.27)	2.92 (0.27)	0.396^£^	2.91 (0.29)	2.91 (0.30)	0.581^£^
Inverse Simpson	10.32 (3.25)	10.01 (3.01)	0.831^£^	10.62 (2.88)	10.12 (3.05)	0.509^£^	10.13 (3.47)	9.91 (2.95)	0.595^£^

^#^Fisher’s exact test, ^£^Independent samples t-test.

^a^Data available for n = 603.

BMI = body mass index, SD = standard deviation.

### Alpha and beta diversity

Alpha diversity (the diversity of microbes within a sample) indexes did not differ between caries and cavity free groups when both sexes were combined (Shannon: *p* = 0.613; Inverse Simpson [Invsim]: *p* = 0.831) (Table 1), indicating that diversity and the richness of the microbial communities were similar between groups (S1 Fig). Microbiota composition in terms of beta diversity (differences in microbial communities between two samples) was determined (Table 2) firstly by sex (*p* = 0.014), secondly by gingival health (*p* = 0.026), and finally by caries (*p* = 0.044) but not by native language (*p* = 0.162). Based on these findings, we performed sex-stratified analyses in order to extract the association of caries status with saliva microbiota in homogenous subsets.

**Table 2:** Determinants of beta diversity in whole group, and separately in boys and girls.

	R^2^	F-value	*p*-value
**Whole group**			
Sex	0.004	2.551	0.014
Gingival health	0.003	2.106	0.026
Caries status	0.003	2.047	0.044
Native language	0.004	1.315	0.164
**Sex-stratified**			
**Boys**			
Caries status	0.007	1.787	0.070
Gingival health	0.006	1.489	0.142
**Girls**			
Caries status	0.004	1.240	0.230
Gingival health	0.005	1.658	0.105

PERMANOVA analysis for the beta diversity using Bray Curtis dissimilarity index among the study participants adjusted for age, gender, BMI z-score, native language, and gingival health.

### Analyses stratified by sex

#### Alpha and beta diversity

**Figure 1:**
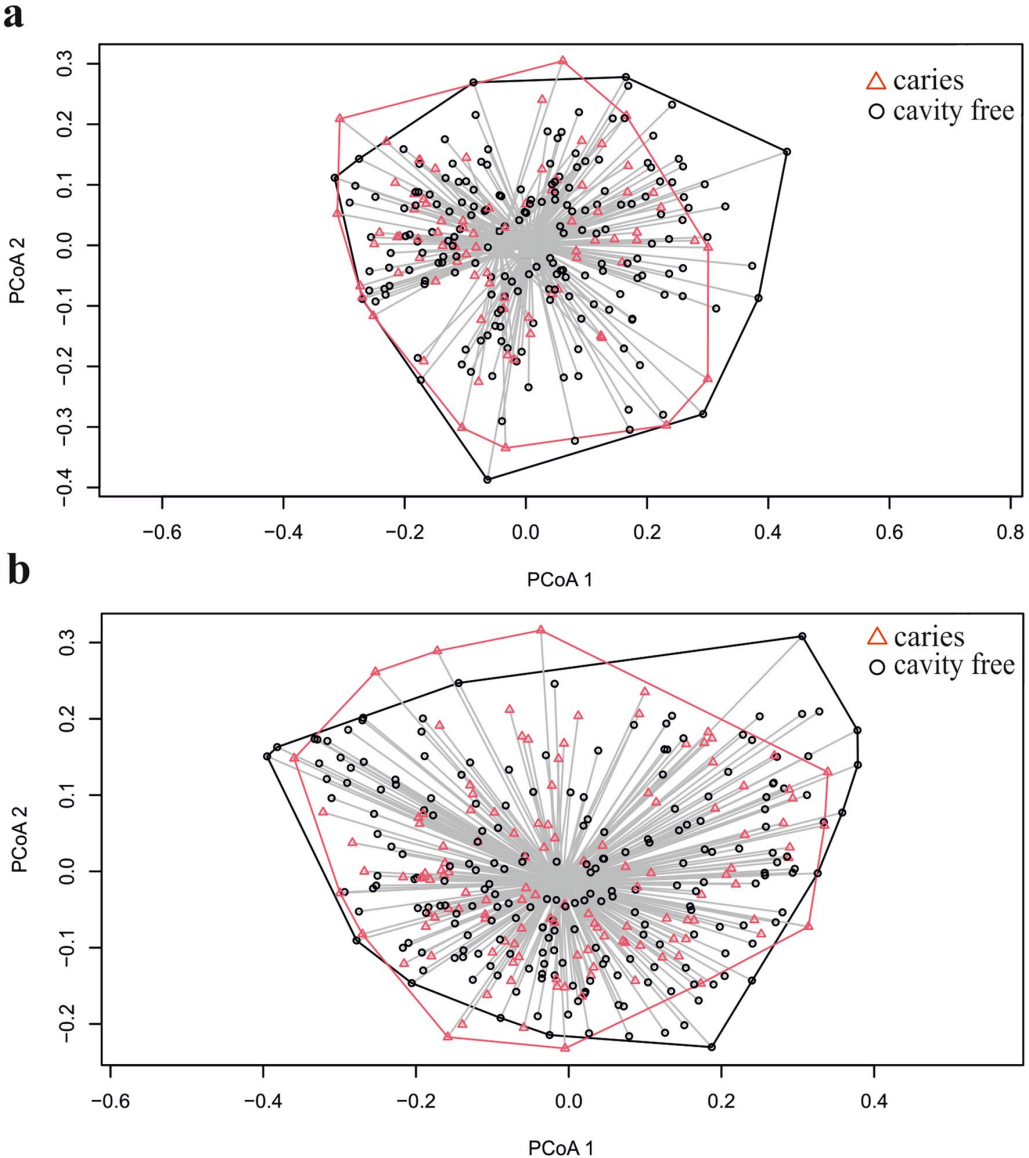
Principal coordinates analysis (PCoA) plot of abundance based on Bray–Curtis dissimilarity (beta diversity) of the saliva microbiota composition in caries and cavity free groups in boys (**a**) and girls (**b**).

We compared alpha diversity indexes by caries status separately in boys and girls. In boys, Shannon and Invsim indexes did not differ between the groups (*p* = 0.396 and *p* = 0.509, respectively) (Table 1). Similarly, in girls, no significant differences in alpha diversity (Shannon: *p* = 0.581; Invsim: *p* = 0.595) were observed between caries and cavity free groups (Table 1). There were no differences in beta diversity between caries and cavity free groups among boys (*p* = 0.070) or among girls (*p* = 0.230) (Fig. 1; Table 2).

### Differential abundance

**Figure 2:**
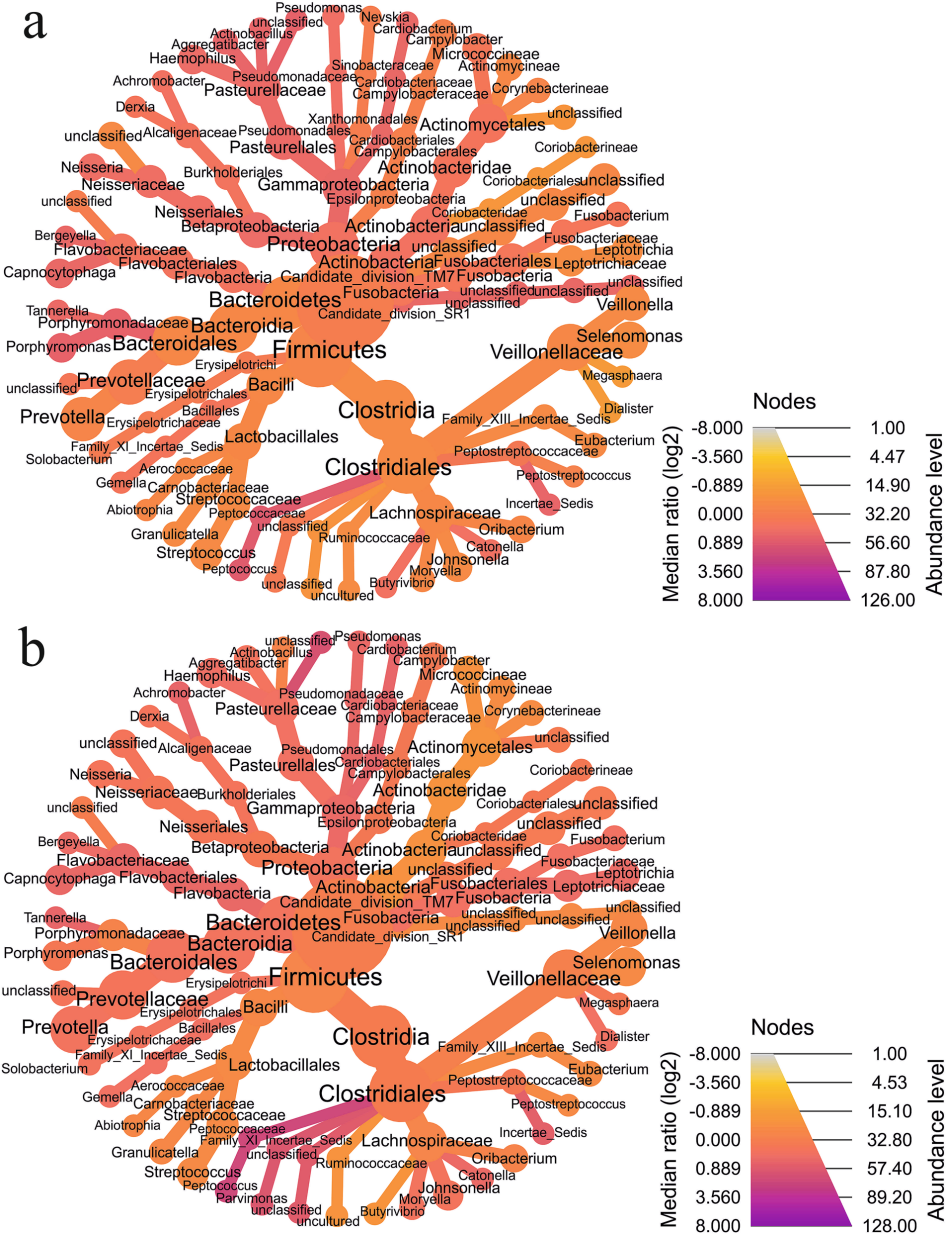
Heat tree analysis leverages the hierarchical structure of taxonomic classifications to quantitatively (using median abundance) and statistically (using non-parametric Wilcoxon Rank Sum test) depict taxonomic differences between microbial communities in boys (**a**) and girls (**b**). The image is drawn using web-based platform MicrobiomeAnalyst^27^ (https://www.microbiomeanalyst.ca/) and trees are automatically created and arranged when there are multiple roots to the taxonomy^28^.

In boys, the five top genera *Rothia, Neisseria, Haemophilus*, *Paludibacter*, and *Labrenzia* were more abundant in the caries group (adjusted *p*-value < 0.001), while *Anaerococcus* (*p* = 0.005)*, Caulobacter* (*p* = 0.004), *Macrococcus* (*p* = 0.004), *Phenylobacterium* (*p* = 0.004)*,* and *Acinetobacter* (*p* = 0.004) were less abundant with caries than cavity free group (Table 3; Fig. 2a).

**Table 3:** List of differentially abundant genera in boys in caries group compared with cavity free group.

OTUs	Genus	log2FoldChange	*p*-value	Adjusted *p*-value
Otu000007	*Rothia*	+ 11.094	0.001	< 0.001
Otu000006	*Neisseria*	+ 10.735	0.001	< 0.001
Otu000008	*Haemophilus*	+ 10.563	< 0.001	< 0.001
Otu000130	*Paludibacter*	+ 1.556	< 0.001	< 0.001
Otu000176	*Labrenzia*	+ 1.189	< 0.001	< 0.001
Otu000222	*Shuttleworthia*	− 1.379	0.010	0.012
Otu000492	*Mogibacterium*	− 3.329	< 0.001	0.003
Otu000349	*Brachymonas*	− 3.964	0.012	0.013
Otu000748	*Selenomonas*	− 4.228	0.005	0.006
Otu004001	*Stenotrophomonas*	− 5.577	0.002	0.004
Otu000804	*Pelomonas*	− 6.145	0.002	0.004
Otu001818	*Acinetobacter*	− 6.173	0.002	0.004
Otu002378	*Phenylobacterium*	− 6.247	0.002	0.004
Otu001497	*Macrococcus*	− 6.353	0.002	0.004
Otu005048	*Caulobacter*	− 6.577	0.002	0.004
Otu005348	*Anaerococcus*	− 7.577	0.004	0.005

log2FoldChange value (+) means higher and (–) means lower abundancy in caries group compared with cavity free group.

Similarly, in girls, the five top genera *Prevotella, Selenomonas, Actinomyces, Paludibacter* and *Labrenzia* were highly abundant in the caries group (adjusted *p*-value < 0.001), while *Hyphomicrobium, Bdellovibrio*, *Weissella*, *Alistipes*, and *Xylanibacter* were less abundant with caries than cavity free group (adjusted *p*-value < 0.001) (Table 4; Fig. 2b).

**Table 4:** List of differentially abundant genera in girls in caries group compared with cavity free group.

OTUs	Genus	log2FoldChange	*p*-value	Adjusted *p*-value
Otu000003	*Prevotella*	+ 11.482	< 0.001	< 0.001
Otu000009	*Selenomonas*	+ 10.168	< 0.001	< 0.001
Otu000033	*Actinomyces*	+ 7.182	< 0.001	< 0.001
Otu000130	*Paludibacter*	+ 1.719	< 0.001	< 0.001
Otu000176	*Labrenzia*	+ 0.853	< 0.001	< 0.001
Otu001039	*Rhodanobacter*	− 3.719	< 0.001	< 0.001
Otu001497	*Macrococcus*	− 4.219	0.006	0.007
Otu000648	*Dolosigranulum*	− 4.249	0.001	0.002
Otu000804	*Pelomonas*	− 4.912	0.002	0.003
Otu002174	*Leuconostoc*	− 6.804	< 0.001	0.001
Otu002378	*Phenylobacterium*	− 6.804	< 0.001	0.001
Otu003167	*Xylanibacter*	− 6.804	< 0.001	0.001
Otu004423	*Alistipes*	− 6.804	< 0.001	0.001
Otu005632	*Weissella*	− 7.554	< 0.001	0.001
Otu005084	*Bdellovibrio*	− 7.564	< 0.001	0.001
Otu004975	*Hyphomicrobium*	− 7.627	< 0.001	0.001

log2FoldChange value (+) means higher and (–) means lower abundancy in caries group compared with cavity free group.

Taken together, the shared saliva microbes found with greater abundancy in both sexes were *Paludibacter* and *Labrenzia* in the caries group than in the cavity free group, while, *Phenylobacterium, Macrococcus* and *Pelomonas* were less abundant. On the other hand, *Selenomonas* showed an inconsistent abundancy in boys and girls.

## Discussion

This study employed 16S rRNA amplicon sequencing to characterize the saliva microbial communities in preadolescent children with and without history of caries in permanent dentition. In total, the history of cavitated caries lesions was recorded in 34% of our participants. Girls were somewhat overrepresented in the caries group. Sex was a stronger determinant of the saliva microbiota than caries. Caries was associated with differences in the abundance of several taxa but not with overall microbial composition or diversity. *Paludibacter* and *Labrenzia* were the key caries-related taxa inhabitants both in preadolescent boys and girls, suggesting a possible pathogenic role of these genera in caries development.

*Paludibacter* is an anaerobic, chemoorganotrophic bacterium in the phylum Bacteroidetes. Recently, this genus was found in the subgingival plaque of periodontally healthy individuals^29^. Similar to other common saliva microbiota (such as *Fusobacterium* and *Gemella*), *Paludibacter* also helps in energy metabolism pathways and cell motility^30^. *Labrenzia* is a genus of bacteria from the phylum Proteobacteria and only a few species have been identified in this genus. *Labrenzia* is mainly found in soil and marine environments^31^. We believe the genus *Labrenzia* has not been linked with caries before. Along with other acid producers, *Labrenzia* are assumed to enhance salivary acidification, which contributes to the progression of dental caries. *Paludibacter* and *Labrenzia* are the two shared saliva microbes positively associated with caries in our study both in boys and girls.

The aetiology of caries is complex and alterations in the saliva microbiota are associated with the development of caries in children^9,23,24^. We observed minor differences in the abundance of several taxa between caries and cavity free groups in sex-stratified analysis. Sex was the major determinant of saliva microbiota composition; we are unaware if previous studies have considered sex in their analysis. Similar to our findings, Jiang et al.^32^ also found minor differences in the abundance of several species in caries and cavity free groups in children (3–4 years old) without formal statistical significance. However, they did not observe any difference in salivary microbiota diversity or composition between boys and girls^32^. In our study, beta diversity was similar between caries and cavity free groups. In subjects 60 years and older, Jiang et al.^22^ observed differences in beta diversity between caries and cavity free groups which is inconsistent with our study^22^. However, most of previous studies included very small sample sizes and were performed at different geographic locations^9,22,24,32^. Evidently, we have conducted the largest study on saliva microbiota and the history of cavitated caries lesions in children.

The sex-stratified analysis revealed potential pathogens, suggesting similar pathogenesis of caries in girls and boys. The timing of dental development differs slightly between boys and girls^33^. However, our results are consistent with other studies that demonstrate that caries is more common in girls than in boys^34,35^. Ortiz et al.^25^ examined the sex-specific differences in the saliva microbiota in children with active caries and identified several species (including *Rothia aeria, Neisseria flavescens*, and *Haemophilus pittmaniae*) that were higher in boys with active caries. Our findings are in line with these observations. While the genus *Rothia* is a key member of the saliva microbiota in persons with caries, its role is largely unknown^36,37^. The majority of the *Neisseria* species are assumed to be acid producers and therefore enhance the development of a cariogenic environment^25^. We found that *Haemophilus* was higher in boys with caries; however, the genus *Haemophilus* was also reported in the saliva microbiota in those with low levels of caries^38^. We found that the genera *Phenylobacterium, Macrococcus* and *Pelomonas* were less abundant in the caries group than in the cavity free group in both sexes. *Phenylobacterium* are strictly aerobic non-motile bacteria that belong to *Caulobacteraceae* family, and the role of *Phenylobacterium* species in saliva microbiota has not been elucidated yet^39^. *Macrococcus* genus belongs to the family Staphylococcaceae, and is closely related to the genus *Staphylococcus,* and this genus is considered nonpathogenic^40^. We have previously reported that *Macrococcus* were less abundant in the saliva of overweight children compared with normal weight children^2^. *Pelomonas* is a genus from the family Comamonadaceae and the abundance of *Pelomonas* has shown to decrease with age in the microbiota of gingival crevicular fluid and tongue back in healthy individuals^41^. Our finding on *Selenomonas* is discordant between sexes. Ortiz et al.^25^ showed that *Selenomonas* sps were associated with caries in girls, and a notably greater abundance of *Selenomonas* Genus probe 1 was observed with less frequent teeth brushing in adolescents with caries^20^, which is in line with our finding in girls. Taken together, we found that most of the caries-associated taxa were acid producers that cause demineralisation of tooth enamel. Accordingly, children with a greater abundance of acid producers may be more prone to caries development.

In addition to oral hygiene, frequent consumption of dietary sugars is the leading cause of caries; bacteria in the oral cavity break down dietary sugars and produce acids that destroy tooth enamel, slowly leading to tooth decay^42^. Our results highlight dissimilarly of saliva microbiota between sexes, while support similar taxa related to cavitated caries lesions. Tooth-brushing and eating habits were not considered here, although they may differ between girls and boys, as reported before ^20,43^. Moreover, variation in the quality of tooth enamel, saliva flow and composition may also contribute to gender differences in saliva microbiota as well as in caries^44,45^. Thus, further studies are needed to address their effects on caries.

The strengths of the present study include a large sample size, the inclusion of a homogeneous age group of both sexes, and availability of primary healthcare visit data collected in dental appointments. Caries was diagnosed by a dental professional and was not self-reported as in many other studies^46,47^. However, our study has several limitations. First, the nature of the study is descriptive due to a cross-sectional study design. From the randomly selected 1000 saliva samples, we limited our material to 617 based on the available records on dental examinations within 12 months. The mean time difference between the saliva sampling and dental appointment was 4.4 months, which we consider a fairly representative timeframe. Our study compared groups with or without history of cavitated caries lesions, not caries activity, in relatively low caries-risk paediatric population, thus a wider timeframe was considered justified. Secondly, participants in the present study were children with late mixed or fully permanent dentition. The eruption schedule of permanent teeth is thoroughly studied and well-known^48^; deciduous teeth are typically exfoliated at 6 to 12 years of age and during this time children have a mixture of permanent and deciduous teeth. Since records on deciduous teeth were limited, the analyses were based on permanent dentition. Hence, early caries experience in deciduous teeth was not included, which may affect our results. Moreover, information on tooth brushing, smoking and other dental hygiene habits is not known.

Dental health measured with the DMFT index has improved among Finnish children since the 1990s^49^. The mean DMFT index values were 0.7 in 12-year-olds in 2009 and our observation is in agreement with this^49^. For our study, the DMFT index was available as a composite variable, which is an indicator of individual’s history of cavitated caries lesions rather than current caries activity. This discrepancy and the overall usability of the DMFT index has been widely discussed^50^, and more elaborate indexes have been suggested, such as the T-Health index^51^. For periodontal or gingival health status, information was obtainable solely as CPITN index scores. Since actual diseases of the periodontium are rare in this age group, we dichotomized the variable and considered it as a reflection of the level of dental hygiene. Finally, our microbiota analysis was limited to the genus level. However, species-level identification would provide a more precise identity in terms of potential biomarkers related to caries. We demonstrated that *Paludibacter* and *Labrenzia* are the ‘keystone pathogens’ that influence the caries process by altering the ‘healthy’ microbiota to a disease state. Such observation have also been reported in periodontal disease^52^.

To conclude, cavitated caries lesions in permanent dentition is associated with relatively minor changes in the saliva microbiota in preadolescents with relatively good dental health. We identified *Paludibacter* and *Labrenzia* as potential biomarkers of caries; both are sugar metabolisers. *Paludibacter* and *Labrenzia* likely enhance salivary acidification, which contributes to the progression of dental caries. However, the clinical relevance of our findings warrants further studies.

## Materials and methods

### Study design

This study utilized saliva samples from the Finnish Health in Teens (Fin-HIT) study, which includes approximately 11,400 children aged mainly 9–12 years. Data were collected between 2011 and 2014 mostly in schools across Finland. The study protocol has been described in detail elsewhere^53^. Altogether 1000 samples were randomly selected from this cohort to obtain an unbiased representation of the population. Age and native language (Finnish, Swedish or other) were reported by the participants or their parents. BMI z-score was calculated based on measured weight and height. Participants with antibiotic use during the 3 months prior to sampling^54^ (n = 31) or without sufficient information on oral health (n = 255) were excluded. After saliva processing, 25 participants withdrew their consent. Furthermore, after processing the microbiota we found that 72 samples had low sequence depth, which may influence the diversity (< 10,000 sequences). These samples were also excluded. The Fin-HIT study protocol was approved by the Coordinating Ethics Committee of the Hospital District of Helsinki and Uusimaa in Finland (169/13/03/00/10). Written informed consent was obtained from the children and one of their parents. All study procedures were performed in accordance with the Helsinki Declaration.

### Oral health

Data on oral health variables were collected from Avohilmo maintained by the Finnish Institute for Health and Welfare (THL) (https://thl.fi/en/web/thlfi-en). Variables regarding the prevalence and history of dental caries were scores of decayed (D) permanent teeth, missing (M) permanent teeth due to caries, and filled (F) permanent teeth. The information on caries status was collected from dental appointments closest to the saliva sampling within 12 months. The mean time difference between the saliva sample collection and dentist appointment was 4.4 (SD 2.25) months. The DMFT index was summarised based on caries status scores in permanent dentition^50,55^, and the composite DMFT index was used as the main outcome. Children were classified into the following two groups: DMFT value ≥ 1 was considered as ‘caries’ and DMFT = 0 as ‘cavity free’. The final study population consisted of 617 participants (n = 208 for caries and n = 409 for cavity free).

Gingival health status was registered in Avohilmo records with the CPITN as reported by Ainamo et al.^56^; this was used as an indicator of dental hygiene status. We categorized the study participants in two groups by their highest CPITN value; CPITN value = 0 was considered as ‘no oral hygiene risk’ and CPITN value 1–2 indicated ‘elevated risk regarding oral hygiene’.

### 16S rRNA gene sequencing and processing of sequencing data

We have previously reported associations of saliva microbiota composition and diversity with weight status^2^, meal regularity^57^, breast feeding^58^, and lifelong antimicrobial purchases^54^. Saliva samples (unstimulated, up to 2 ml) were collected from each participant in Oragene-DNA (OG-500) self-collection kits (DNA Genotek Inc., Canada) containing a stabilizing reagent and transported to the laboratory for DNA extraction. The samples were lysed using 50 ml lysozyme (10 mg/ml, Sigma-Aldrich), 6 ml mutanolysin (25 KU/ml, Sigma-Aldrich), and 3 ml lysostaphin (4000 U/ml, Sigma-Aldrich), after which a 500-ml aliquot of cell suspension was added and further incubated for 1 h at 37 °C. After lysis, genomic DNA was extracted using a CMG-1035 saliva kit and Chemagic MSM1 nucleic acid extraction robot (PerkinElmer)^2,54^. 16S rRNA amplification was performed with an in-house protocol as described previously^59^. The V3—V4 variable regions were amplified using primers reported previously^60^. DNA quantity was assessed using an Agilent 2100 Bioanalyzer and PCR products were processed for paired-end sequencing (2 × 270 bp) on a HiSeq1500 platform (Illumina, CA, USA).

The paired-end reads were merged together to reconstruct full-length sequences using mothur pipeline (Version v.1.35.1). Sequencing quality was performed and further processed using the MiSeq SOP^61^. The sequence reads containing ambiguous bases (N), homopolymer stretches (> 8 bases), and small reads (< 330 bases) were removed using mothur pipeline^62^. UCHIME algorithm incorporated in the mothur was used to remove chimeric sequences^63^. The high-quality sequence reads were then aligned to the Silva 16S rRNA reference database^64^ (Ver V119) and clustered at > 98% homology to identify Operational Taxonomic Units (OTUs). The most abundant bacterial taxa were recognized at the genus level. Alpha diversity indexes (Shannon index and Invsim index) were calculated to illustrate the microbiota diversity and richness in each sample. Beta diversity was calculated using Bray Curtis dissimilarity index. These were performed using the R-package ‘vegan’ (version 2.5-6)^65^.

### Statistical analysis

The normality of the distributions was visually examined, and appropriate tests were used for the analysis. Independent samples t-test (for continuous variables) and Fisher’s exact test (for categorical variables) were performed to examine differences between the caries and cavity free groups. Permutational analysis of variance (PERMANOVA) test was used to test the differences in microbial community composition between individuals with caries vs cavity free using the adonis and betadisper function in Phyloseq R-package (version 1.32.0). Differentially abundant OTUs were identified at the genus taxonomic level using DESeq2 incorporated in Phyloseq R-package (version 1.32.0). The results are presented with mean (SD) unless indicated otherwise. All statistical analyses were conducted using IBM SPSS for Windows, version 20 (IBM, Chicago, IL, USA). The statistical significance level was set at 5%. *p*-values were calculated and adjusted by the false discovery rate (FDR).

## Supplementary information

Supplementary Figure.

## Data Availability

All relevant data are within the manuscript and its Supporting Information files.
