# Supplementary figures and images for: High abundance of sugar metabolisers in saliva of children with caries
Source: Sci Rep. 2021 Feb 24;11:4424. doi: 10.1038/s41598-021-83846-1 (PMC7904847; doi:10.1038/s41598-021-83846-1)

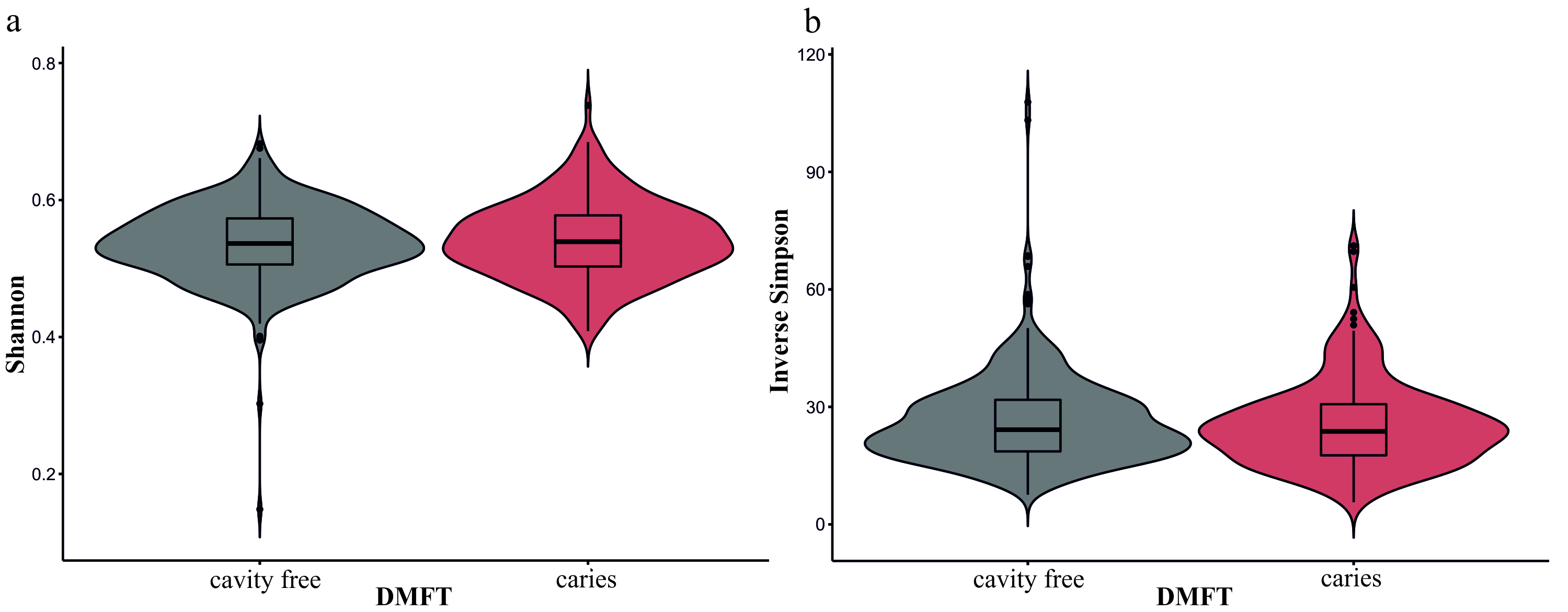

Supplement: Supplementary file 1 — Supplementary Figure. [file 41598_2021_83846_MOESM1_ESM.jpg]
